# Factors influencing adherence to lifestyle prescriptions among patients with nonalcoholic fatty liver disease: A qualitative study using the health action process approach framework

**DOI:** 10.3389/fpubh.2023.1131827

**Published:** 2023-03-17

**Authors:** Lina Wang, Huixuan Zhou, Yali Liu, Xin Wang, Wenjing Yan, Jing Zhang, Hong Ren

**Affiliations:** ^1^Department of Physical Fitness and Health, School of Sport Science, Beijing Sport University, Beijing, China; ^2^The Third Unit, Department of Hepatology, Beijing Youan Hospital, Capital Medical University, Beijing, China; ^3^Department of Nursing, Beijing Youan Hospital, Capital Medical University, Beijing, China; ^4^School of Physical Education, Shanxi Normal University, Taiyuan, China

**Keywords:** nonalcoholic fatty liver disease (NAFLD), Health Action Process Approach (HAPA), lifestyle, exercise, diet, physical activity

## Abstract

**Background and objective:**

Lifestyle modifications aimed at weight loss have been introduced as a cornerstone of nonalcoholic fatty liver disease (NAFLD) management. However, very few patients follow the doctor's prescription to change their lifestyle to achieve weight loss in the real world. The purpose of this study was to use the Health Action Process Approach (HAPA) model to examine the factors that affect adherence to lifestyle prescriptions among patients with NAFLD.

**Methods:**

Semi-structured interviews were conducted with patients with NAFLD. Reflexive thematic analysis and framework analysis were used to determine naturally identified themes and allocate them to theoretically driven domains.

**Results:**

Thirty adult patients with NAFLD were interviewed, and the identified themes were mapped directly onto the constructs of the HAPA model. This study revealed that key barriers to adhering to lifestyle prescriptions are related to the coping strategy and outcome expectation constructs of the HAPA model. For physical activity, conditional limits, lack of time, symptoms such as fatigue and poor physical fitness, and fear of sports injury are the primary barriers. Barriers to diet are mainly diet environment, mental stress, and food cravings. Key facilitators for adherence to lifestyle prescriptions include developing simple and specific action plans, coping strategies to flexibly deal with obstacles and difficulties, receiving regular feedback from doctors to improve self-efficacy, and using regular tests and behavior recording to enhance action control.

**Conclusions:**

Future lifestyle intervention programs should pay particular attention to the planning, self-efficacy, and action control-related constructors of the HAPA model to promote the adherence of patients with NAFLD to lifestyle prescriptions.

## 1. Introduction

Nonalcoholic fatty liver disease (NAFLD) is the leading cause of chronic liver disease worldwide, affecting 25% of the global population. NAFLD is associated with cardiometabolic disorders: obesity, insulin resistance, type 2 diabetes mellitus, high blood pressure, and atherogenic dyslipidemia, all of which increase the risk of a heart attack or stroke (1). Due to the close relationship between NAFLD and metabolic syndrome, some scholars have recently proposed renaming NAFLD as metabolic-associated fatty liver disease (MAFLD) (2). The prevalence of NAFLD has increased to alarming levels, placing an enormous burden on affected individuals and healthcare systems (3).

Lifestyle modifications aimed at weight loss have been introduced as a cornerstone of NAFLD management. However, it is difficult for patients with NAFLD to change their lifestyle to achieve weight loss in the real world. A study showed that only 30% of patients with NAFLD lose more than 5% of their body weight within 13 months (4). NAFLD clinical guidelines reported that lifestyle intervention programs should be recommended, when possible, and tailored to patients' preferences to ensure long-term adherence (1, 3, 5, 6). Currently, there is limited research on the factors affecting adherence to lifestyle prescriptions among patients with NAFLD. To the best of our knowledge, there is only one study that has explored the preferences and unmet needs of individuals with nonalcoholic steatohepatitis (NASH) (7). The study pointed out that patients needed assistance in managing their lifestyles, but it was “not” examined this aspect in depth.

The behavior change model is a theoretical psychological method of describing the factors that affect in all kind of behavior, which can guide the development of behavior intervention (8). One such model is the Health Action Process Approach (HAPA) (9). The HAPA model postulates that behavior change is a dynamic and continuous process comprising two distinctive phases. The first phase is the motivational phase, which plays an important role in increasing risk perception (e.g., the risk of NAFLD disease becoming serious) and action self-efficacy (e.g., how to stay confident in the ability to sufficiently perform regular exercise and a healthy diet plan), and managing outcome expectations (e.g., expected pros and cons of the outcomes of participating in changing an unhealthy lifestyle); this strengthens the formation of intentions for behavior change (e.g., “I intend to do physical activities at least 5 days per week with 30 min each time”).

Once individuals have established an intention, they enter the second phase, the volitional phase (10). In this phase, individuals may benefit from a series of volitional self-regulation strategies for behavior initiation and maintenance. This includes action plans (e.g., when, where, and how to enact regular exercise), coping strategies (e.g., how to maintain a limited energy intake level when confronted with obstacles), maintenance self-efficacy (e.g., how to stay confident about the ability to eat sufficient portions of fruits and vegetables when obstacles occur), recovery self-efficacy (e.g., how to stay confident about the ability to restart exercise behavior after disengagement), and action control (e.g., how to constantly self-monitor themselves to prevent relapse). In addition, promoting an individual's perceived social support is equally important for maintaining behavior and preventing relapse (11).

The HAPA model has been widely used in various areas of behavior change, including interventions for diet (12), physical activity (13), and meditation adherence (14). Some studies have shown a strong correlation between the HAPA model and improved diet and exercise adherence in individuals with chronic diseases (15), suggesting that the HAPA model is appropriate for developing adherence to lifestyle prescriptions. To the best of our knowledge, to date, only one qualitative study has explored the application of the HAPA model to pharmacist-assisted medication adherence among unreachable patients (16). How to use HAPA model to guide patients with chronic diseases to adhere to a healthy lifestyle is still worth studying.

Hence, this study aimed to investigate the factors influencing adherence to lifestyle prescriptions aimed at weight loss within the framework of HAPA so as to provide ideas for lifestyle intervention for patients with NAFLD and other chronic diseases.

## 2. Methods

### 2.1. Study design

This is a qualitative, descriptive study. Conducting individual interviews using thematic analysis was the most suitable approach to understanding the preferences and needs of the patients with NAFLD to adhere to lifestyle prescriptions in a real-world setting. Researcher reflexivity is known to influence the results, as researchers' beliefs, experiences, and context can shape the interpretation of the findings.

The study was conducted from July 2022 to September 2022. The study was conducted in accordance with the ethical guidelines of the 1975 Declaration of Helsinki and was approved by the Ethical Committee of Beijing Youan Hospital (approval number: 2018–095). All patients provided written informed consent to have their information used (anonymously) for research purposes.

### 2.2. Participants

Patients with NAFLD were recruited from the outpatient clinic of Beijing Youan Hospital. The inclusion criteria were as follows: (1) aged above 18 years and (2) diagnosed with fatty liver by B-type or liver biopsy. The exclusion criteria were as follows: (1) having hepatitis B or C virus and (2) having a diagnosis of liver cirrhosis or hepatocellular carcinoma. We employed a purposive sampling strategy with maximal variation to identify shared patterns in the data (17). We aimed to recruit a sample of patients based on their age, gender, and length of time since diagnosis. It was estimated that 30 interviews would be needed to reach thematic saturation (as determined by an investigator consensus, as is the gold standard in qualitative studies) (18).

### 2.3. NAFLD definition and classification

The presence of fatty liver disease of all patients involved was assessed using a B-type ultrasound. The presence of fatty liver disease of some patients were also confirmed using liver biopsy. Liver fat content and the presence of fibrosis were measured with the Fibroscan 502 touch device (Echosens, Paris, France). Regarding the value of the controlled attenuated parameter (CAP), liver steatosis was classified into three grades: mild liver steatosis: 238–258 db/m, moderate liver steatosis: 259–292 db/m, and severe liver steatosis: >292 db/m. The degree of liver steatosis corresponded to the degree of fatty liver.

### 2.4. Procedures

The hepatologist referred the eligible patients to a research assistant in the adjacent room, who then explained the purpose of the study to them. Those who were interested were provided with a detailed explanation of the study and the interview process. Only those who were willing to participate were provided with consent forms and then proceeded with the interview. Thirty patients were referred to the research assistant, and all of them agreed to be interviewed.

The eligible participants participated in a semi-structured individual face-to-face interview for approximately 10–40 min each. The interviews were conducted by a member of the research team with expertise in qualitative research. The HAPA framework was used to develop the interview schedule. The interview questions (see Appendix Interview Guide) were based on the participants' understanding of the role of a healthy lifestyle and its effects on NAFLD, the outcomes of inactivity and an unhealthy diet, the need for a lifestyle intervention plan, the difficulties and obstacles in implementation, the need for coping strategies, ways to improve the ability of action control and self-efficacy, and so on. The interviews were transcribed verbatim and modified to remove any identifying information. The transcripts were then uploaded to NVivo11 (QSR International, Melbourne, Australia) qualitative analysis software.

### 2.5. Analysis

Both reflexive thematic analysis and framework analysis were used to determine naturally identified themes and then allocate them to pre-selected theoretically driven domains to assist in the identification of the factors affecting adherence to lifestyle prescriptions; we followed Braun and Clarkes' (19) instructions for conducting a reflexive thematic analysis. First, two researchers, L.W. and H.Z., became familiar with the interview material after multiple readings of the transcripts (Step 1, Familiarization). This included note-taking to help facilitate proper comprehension of the data. Second, subsequently, L.W. and H.Z. independently coded each interview (Step 2, Generating codes). An inductive thematic analysis was conducted to identify new “candidate” themes by combining similar codes to create major categories using a thematic map (Step 3: Constructing themes). These themes were then deductively mapped against the HAPA (9). These themes were then reviewed and checked against the data set, and “candidate” theme names were provided, clearly reflecting the meaning of each (Steps 4 and 5, Revising and defining themes). Finally, the results were written down, which allowed the continuous testing of the themes (and refinement if needed as a final stage of analysis) to determine how well they answered the research question. The quotes were anonymized and presented to illustrate the core meaning of the themes (Step 6, Producing the report).

### 2.6. Reflexivity

L.W. conducted qualitative interviews with the participants. L.W. is a female research fellow with experience in qualitative research in lifestyle management. This may have influenced the questions that the interviewer delved further into, for example, when the participants spoke about the need for a lifestyle plan. The researchers involved in the analysis (L.W. and H.Z.) are accredited exercise physiologists and dietitians, indicating that they brought a wealth of knowledge to the process of data interpretation. While this may have affected the interpretation of the results, the themes were deductively mapped to the pre-established domains. The remaining authors were not involved in the qualitative interviews or data analysis; however, they were allowed to review themes and suggest different interpretations of the data.

## 3. Results

### 3.1. Participant characteristics

Table 1 displays the characteristics of the 30 participants. The age of participants ranged from 22 to 63 years, with a mean age of 40.2 years (SD = 11.8). Half of the participants were women, and 30.0% of them were unmarried. Additionally, 80.0% of the participants had an undergraduate level of education or above. All the patients involved had severe fatty liver disease.

**Table 1:** Participants' characteristics.

**Variables**		***n* (%)**
Age	<30	5 (16.7)
	30–39	9 (30.0)
	40–49	8 (26.7)
	≥50	8 (26.7)
Gender Marital status	Male	15 (50.0)
	Female	15 (50.0)
	Married	21 (70.0)
	Unmarried	9 (30.0)
Education level	≤Senior high school level	6 (20.0)
	Undergraduate level	18 (60.0)
	Postgraduate level	6 (20.0)
Weight status	Normal weight	4 (13.3)
	Overweight	14 (46.7)
	Obese	12 (40.0)
Current work status	Working full-time	18 (60.0)
	No work	5 (16.7)
	Student	2 (6.7)
	Retired	5 (16.7)
Monthly income (RMB)	<3,000	9 (30.0)
	3,000–9,000	8 (26.7)
	>9,000	13 (43.3)
Duration of NAFLD	<1 year	3 (10.0)
	1–4 years	10 (33.3)
	5–9 years	9 (30.0)
	≥10 years	8 (26.7)
Commodities	Diabetes	7 (23.3)
	Hypertension	4 (13.3)
	Hyperuricemia	7 (23.3)
	Dyslipidemia	22 (73.3)

### 3.2. HAPA

**Figure 1:**
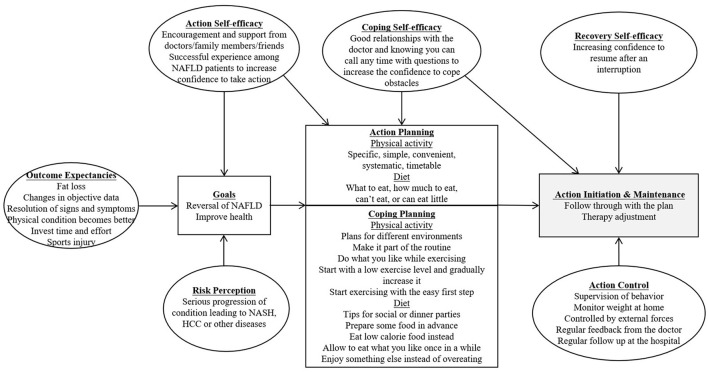
HAPA Framework. The HAPA component of this figure was adapted from Schwarzer (9).

The thematic analysis of the interviews produced multiple themes for each domain (Table 2). The framework was updated based on the thematic data (Figure 1).

**Table 2:** Interview themes.

**Domain**	**Theme**
Risk perception	Serious progression of condition leading to NASH or HCC
	Serious progression of condition leading to other diseases
Outcome expectancies	Fat loss
	Changes in objective data
	Resolution of signs and symptoms
	Physical condition becomes better
	Sports injury
Goals	Reversal of NAFLD
	Improve health
Action plans	Simple
	Convenient
	Specific
	Systematic
	Timetable
Coping strategies	Plans for different conditions
	Make it part of the routine
	Start with a low exercise level and gradually increase it
	Take the first step to start exercising
	Tips for social and dinner parties
	Prepare some food in advance
	Eat some low calorie food instead
	Allow eating what you like once in a while
	Enjoy something else instead food
Action control	Self-supervision of behavior
	Monitor simple indicators at home
	Controlled by external forces
	Regular feedback from the doctor
	Regular follow up at the hospital
Self-efficacy	Encouragement and support from doctors
	Encouragement and support from family members/friends
	Mutual supervision and successful experience among patients

Themes from interview analysis grouped by domain.

### 3.3. Risk perception

Regarding the risk of fatty liver disease, most of the patients were aware that fatty liver disease would progress into cirrhosis and hepatocellular carcinoma, but the majority of the patients were not aware that fatty liver disease would cause other diseases, such as cardiovascular and cerebrovascular diseases. While discussing the impact of an unhealthy lifestyle, most patients reported that the most immediate impact is gastrointestinal discomfort resulting from unhealthy eating habits, which can, in turn, affect the quality of sleep and other aspects of daily life. In addition, regarding long-term effects, most patients stated that an unhealthy lifestyle would aggravate the fatty liver disease and increase the risk of other chronic diseases such as high blood sugar and high uric acid, and so on. The patients with a shorter history of the disease reported higher levels of risk awareness and more frequent unhealthy lifestyle behaviors compared to those with a longer history of the disease.

“Fatty liver will cause cirrhosis. Fatty liver will cause abnormal liver function, which may make me more prone to cancer. I'm worried about that.” P2

### 3.4. Outcome expectancies

Outcome expectancies are divided into positive outcome expectations and negative outcome expectations. While discussing the positive outcomes of a healthy lifestyle, most patients expressed the great importance of treating fatty liver through lifestyle changes. The majority of the patients had experienced the benefits of a healthy lifestyle, such as improved sleep, physical relaxation, greater flexibility, and mental wellbeing. To treat fatty liver disease, the patients expressed their hope to improve clinical indicators and symptoms, such as fatigue, and reduce body fat. In addition, the majority of patients had experienced a relapse of fatty liver disease. They expressed a wish for this disease's symptoms to be resolved. Regarding the possible negative outcomes of a healthy lifestyle, the patients expressed concerns about potential sports injuries resulting from engaging in regular exercise. These concerns served as a barrier to patients' willingness to exercise regularly. Regular exercise as a treatment for fatty liver is associated with a negative outcome expectancy, which is sports injury. Most patients who were overweight or obese were worried about knee joint injuries, which hindered their ability to adhere to regular exercise for a long time.

“I went out for a walk before and felt relaxed. I slept soundly at night.” P1

“There is repeated experience of fatty liver disease. If I pay attention to the lifestyle, the fatty liver disease will improve; if I don't pay attention to the lifestyle, the fatty liver disease will get worse.” P17

### 3.5. Goals

The majority of patients stated they did not have a clear goal for fatty liver treatment. They anticipated that the fatty liver would resolve itself and not become worse. The patients were aware that they needed to exercise more and eat less as part of their lifestyle management goals; however, they did not establish specific goals. Moreover, although most patients had experienced weight loss, they had not set clear goals for their weight management.

“I know I need to eat less and exercise more. I don't know how.” P10

### 3.6. Action plans

#### 3.6.1. Physical activity

Most patients knew that they needed to engage in more physical activity, but they had not made or provided a detailed plan. They expected simple, convenient, specific, and systematic timetable-based plans. Some patients reported that creating a detailed plan would make implementing and adhering to lifestyle prescriptions easier.

#### 3.6.2. Diet

The patients acknowledged the need to reduce their food quantity, eat more vegetables, and reduce their intake of staple foods and oil, among other things, but they did not know how to arrange their diet to fit their needs: What to eat, how much to eat, or what type of food they need to eat. A recipe or dietary standard may be helpful. Some patients mentioned finding it difficult to estimate the weight of their food and expressed a desire for a more understandable method of measurement.

Most patients reported receiving exercise and diet advice from other liver doctors or healthcare professionals, but the advice they received was general and lacked specificity. Moreover, when the patients looked up relevant knowledge, they found it difficult to differentiate between right and wrong information. The patients with NAFLD expressed a desire for professional lifestyle management to help them manage their condition more effectively.

“I didn't make a plan. I went to exercise if I wanted to, and I didn't go if I didn't want to.” P18

“Just to know how should I eat every day, I want to get a nutrition plan that will meet my nutritional needs without having excess energy.” P23

### 3.7. Coping strategies

All the patients did not know how to overcome the difficulties and obstacles hindering their adherence to lifestyle prescriptions. While discussing factors that might affect patients' adherence to lifestyle prescriptions, patients reported that the difficulties and obstacles encountered in regular exercise mainly existed in the following areas: (1) conditional limitations, such as adverse weather (rain, cold, and hot), poor air quality, and quarantine at home due to the COVID-19 pandemic; (2) lack of time owing to work commitments, a heavy academic burden, or busy housework, among other things; (3) disease symptoms such as fatigue; and (4) physical fitness, as patients were prone to experiencing fatigue. Notably, women were more frequently hindered from regular exercise than men due to housework. Most patients with NAFLD reported that they had not used coping strategies before and that it was easy for them to give up when encountering these difficulties and obstacles. Having an alternative plan in mind for these situations could help them stick to their exercise routine.

The difficulties and obstacles when attempting to adhere to a diet plan mainly exist in the following aspects: (1) diet environments, such as social and dinner parties or regular meals with few choices; and (2) psychological factors, such as mental stress, hunger cravings, craving for delicious food, and so on. Patients stated that a diet plan that predicts these conditions can help them develop healthy eating habits.

“For example, like last year, I kept running for a while, but it was too cold in winter, and I didn't want to go out for exercise.” P4

“I usually have a lot of learning tasks, which may limit my exercising time. I think maybe I can set an alarm clock and improve my learning efficiency so that I can free up time to exercise.” P3

### 3.8. Action control

The patients stated that the following strategies might help them maintain a healthy lifestyle: (1) self-supervision of behavior through the use of activity trackers or taking photographs of their food; (2) monitoring simple indicators at home, such as weight or waist circumference; (3) seeking external support from others, such as doctors, family members, and others; (4) receiving regular feedback from their doctors to assess whether what they did was right or wrong; and (5) attending regular follow-up at the hospital to monitor changes in their clinical indicators.

“There was a time when I socialized a lot. I gained weight. Well, I can make changes later in time.” P4

### 3.9. Self-efficacy

Self-efficacy is a factor that affects patients' adherence to lifestyle prescriptions. The patients mentioned that the following aspects could improve their self-efficacy and enhance their confidence in modifying their lifestyle: (1) receiving encouragement and support from doctors (being able to contact their doctor whenever they have a problem and receiving their feedback on the effectiveness of their implementation); (2) receiving encouragement and support from family members and friends (verbal encouragement or support through actions); (3) sharing mutual supervision and successful experience with other patients.

“I sometimes go swimming with my friends. We can swim in competitions, which makes it easier for me to keep going. But if my friends don't go, I will also be lazy.” P2

## 4. Discussion

The present study used the HAPA model to identify the factors affecting adherence to lifestyle prescriptions among patients with NAFLD. The main findings of this study were those patients with NAFLD are aware that they can manage their condition by changing their lifestyle, but they are uncertain about what specific actions to take or how to implement them effectively. Patients need to have a strong sense of self-efficacy to manage their condition effectively; they need to have achievable action plans and coping strategies, as well as support networks and methods of action control to facilitate their transition to a healthy lifestyle. This study highlights the significance of action plans, coping strategies, action control, and the self-efficacy construct. These findings have important implications for the design of lifestyle prescriptions that can help patients manage their conditions successfully.

The study was performed in the outpatient clinic of a hepatology hospital. All the patients involved suffered from severe liver disease. Most patients with NAFLD visited the hospital for treatment because of fatty liver disease. At ordinary times, most patients in our clinic had severe fatty liver disease. They received advice on mainly exercise and diet from liver doctors or other healthcare professionals. In addition, 80.0% of the participants had an undergraduate level of education or higher. While the patients' education level in this study was higher than that reported in previous studies involving patients with NAFLD (7, 20, 21), it was similar to the findings that higher education levels are usually associated with greater risk awareness and a greater emphasis on disease treatment (22, 23).

In terms of adhering to regular exercise, having coping strategies is necessary for patients to overcome any obstacles they may encounter while implementing exercise prescriptions. Conditional limitations, a lack of time, and fatigue were reported as barriers to regular exercise in other similar studies (20, 24, 25). Our study also found poor physical fitness as a barrier for patients with NAFLD. Moreover, female patients were more likely to be hindered in their ability to exercise regularly due to household responsibilities. Currently, the research on effective intervention strategies for addressing these barriers is very limited. Some patients with NAFLD also mentioned fatigue or laziness as barriers to following a healthy lifestyle (24). When faced with this situation, taking the first step toward exercising may be helpful, such as putting on sneakers and going outdoors. A few patients mentioned struggling with poor physical fitness and ability; this reason was supported by some studies showing that patients with NAFLD have a lower physical function and performance status (26). Additionally, patients with NAFLD experience perceived exertion more frequently when exercising at similar intensities to those with chronic hepatitis C (27). However, most of them can accept starting with a low level of exercise and gradually increasing it to overcome this barrier (28).

To enhance patients' adherence to regular exercise, this study suggests that action plans should be simple, convenient, specific, and systematic, as well as include timetables. This finding is unsurprising, given that patients desire plans that can be implemented without requiring much thought; these findings are consistent with a similar study conducted on patients with diabetes (28). Although clinical guidelines recommend patients with NAFLD should take aerobic exercise ≥3 days/week (≥150 min/week moderate intensity), resistance exercise ≥2 days/week, and reduce sedentary behavior (29), these reference estimates can support exercise plans, but the planning should be tailored to individual patient's needs, preferences, and access to resources. ACSM's guideline (30) suggests that exercise plans should include frequency, intensity, time, type, volume, and progression. In addition, the content of the plan should be simple, convenient, and easy to carry out, and the format should be a schedule with exact timing.

In terms of diet, the most significant barriers identified were environment and psychological factors, which must be considered when devising coping strategies. The most commonly reported barriers to a healthy diet among the participants in our study were related to the social environment, such as dinner parties or regular meals with limited food options. This finding is similar to that of an Indian study (25), which found that festivals, weddings, family or friend visits, and being provided with meals at the workplace were significant barriers. To address these challenges, recommendations could include reducing participation in social gatherings, eating at home more often, or making conscious food choices when attending dinner parties.

Regarding action plans for a healthy diet, a recipe that includes what to eat and how much to eat would be helpful for patients. Taking pictures or making videos can help patients better understand diet plans. Clinical guidelines recommend appropriate dietary patterns and composition, and dietary habits should be altered primarily based on energy restriction to a daily rate of 500 to 1,000 kcal. Moreover, it has been reported that the amount of weight loss should be the same regardless of the type of diet followed (29). On this basis, clinical practice should be easier to understand and implement.

Self-efficacy has been demonstrated as an important factor for behavior change (31), and our study is not an exception. Our finding further emphasizes that regular motivational support from health workers such as specialists/dietitians/exercise physiologists in patients' lifestyle behavior management is highly beneficial for patients to continue with a healthier lifestyle (7, 32). Patients with NAFLD often lack the motivation to change their behaviors (33). In such cases, patients can contact their doctors when they have questions and receive feedback when they implement the plan, which would be suggested to enhance their confidence.

Action control is a behavioral influence factor that deserves more attention. Action control is similar to self-supervision. Participants demonstrated that their action control was poor and needed to be strengthened. Monitoring of diet and physical activity progress by a healthcare professional was reported to be beneficial. In terms of regular exercise, patients suggested that a pedometer would be a useful tool to allow them to check and monitor their progress (20). In addition, patients can get regular supervision and feedback from doctors. They can also regularly supervise their exercise and diet behavior and monitor weight by themselves. Regular follow-ups by hospitals would help patients maintain a healthy lifestyle.

There are several limitations to note for this study. First, the demographics of the patients involved were fairly homogeneous. All of our patients had severe fatty liver disease, and the average level of formal education was higher than that of the general population. These characteristics could have impacted the reported outcome expectations and risk perceptions, as other studies have highlighted a misunderstanding of fatty liver disease and neglect of treatment. The sociodemographic characteristics of our participants may have also impacted the need for lifestyle prescriptions. Second, the factors we identified from discussions with patients were verified in our study. Therefore, more experiments are needed to examine these factors in the future. Third, the findings do not indicate what made participants behave in one way or another. In the future, researchers should consider studying the path of behavior formation by combining quantitative studies.

Several strengths of this study are also worth considering. One strength of our study was that it is one of the few qualitative studies that focused on acquiring patients' perspectives regarding lifestyle prescriptions. The insights gained from our research add to the existing literature, informing future, larger-scale quantitative studies on the facilitators and barriers of interest. The other strength of our study was that it highlights the importance of action plans and coping strategies, which have not been mentioned in previous studies on this topic.

## 5. Conclusions

The present study used a novel method by adopting the HAPA model to explore strategies for promoting adherence to lifestyle prescriptions among patients with NAFLD. In addition, learning from patients' perspectives is essential to accommodate their feedback for future lifestyle interventions for managing NAFLD. Although the barriers and facilitators identified included all constructs of the HAPA model, action plans, coping strategies, action control, and self-efficacy were the most prominent. Developing a patient-appropriate lifestyle action plan, developing coping strategies to overcome obstacles and deal with difficulties, and receiving feedback from physicians on lifestyle implementation are key facilitators for patients with NAFLD to adhere to an appropriate lifestyle prescription. The present study contributes to a growing body of literature that explores techniques for incorporating the patient's viewpoint into early lifestyle interventions and stakeholder discussions regarding NAFLD management. In the future, we should consider conducting intervention research on these strategies to examine their effects.

## Data availability statement

The original contributions presented in the study are included in the article/Supplementary material, further inquiries can be directed to the corresponding authors.

## Ethics statement

This study was approved by the Ethics Committee of Beijing Youan Hospital (Ethics Number: 2018-095). The patients/participants provided their written informed consent to participate in this study. Written informed consent was obtained from the individual(s) for the publication of any potentially identifiable images or data included in this article.

## Author contributions

Conceptualization and funding acquisition: LW, JZ, and HR. Methodology and formal analysis: LW and HZ. Validation: JZ and YL. Investigation: JZ, YL, and XW. Writing—original draft preparation: LW. Writing—review and editing: JZ and HR. Supervision: WY. Project administration: HZ, JZ, and HR. All authors have read and agreed to the published version of the manuscript.

## References

[1] Cusi K Isaacs S Barb D Basu R Caprio S Garvey WT. American association of clinical endocrinology clinical practice guideline for the diagnosis and management of nonalcoholic fatty liver disease in primary care and endocrinology clinical settings: co-sponsored by the American association for the study of liver diseases (Aasld). Endocr Pract. (2022) 28:528–62. 10.1016/j.eprac.2022.03.010 35569886

[2] Eslam M Newsome PN Sarin SK Anstee QM Targher G Romero-Gomez M. A new definition for metabolic dysfunction-associated fatty liver disease: an international expert consensus statement. J Hepatol. (2020) 73:202–9. 10.1016/j.jhep.2020.07.045 32278004

[3] Eslam M Sarin SK Wong VW Fan JG Kawaguchi T Ahn SH. The Asian pacific association for the study of the liver clinical practice guidelines for the diagnosis and management of metabolic associated fatty liver disease. Hepatol Int. (2020) 14:889–919. 10.1007/s12072-020-10094-2 33006093

[4] Vilar-Gomez E Martinez-Perez Y Calzadilla-Bertot L Torres-Gonzalez A Gra-Oramas B Gonzalez-Fabian L. Weight loss through lifestyle modification significantly reduces features of nonalcoholic steatohepatitis. Gastroenterology. (2015) 149:367–78 e5. 10.1053/j.gastro.2015.04.005 25865049

[5] European Association for the Study of the L, European Association for the Study of D, European Association for the Study of O. Easl-Easd-Easo clinical practice guidelines for the management of nonalcoholic fatty liver disease. J Hepatol. (2016) 64:1388–402. 10.1016/j.jhep.2015.11.004 27062661

[6] Fan JG Wei L Zhuang H National Workshop on Fatty L Alcoholic Liver Disease CSoHCMA Fatty Liver Disease Expert Committee CMDA. Guidelines of prevention and treatment of nonalcoholic fatty liver disease (2018, China). J Dig Dis. (2019) 20:163–73. 10.1111/1751-2980.12685 30444584

[7] Cook NS Nagar SH Jain A Balp MM Mayländer M Weiss O. Understanding patient preferences and unmet needs in nonalcoholic steatohepatitis (Nash): insights from a qualitative online bulletin board study. Adv Ther. (2019) 36:478–91. 10.1007/s12325-018-0856-0 30547371 PMC6824346

[8] Glanz KR Barbara K Viswanath K Orleans C. Tracy. Health Behavior and Health Education 4^th^ ed. San Francisco, CA: Jossey-Bass. (2008).

[9] Schwarzer R. Health action process approach (Hapa) as a theoretical framework to understand behavior change. Actualidades en Psicologí*a*. (2016) 30:119–30. 10.15517/ap.v30i121.23458

[10] Schwarzer R. Modeling health behavior change: how to predict and modify the adoption and maintenance of health behaviors. Appl Psychol. (2008) 57:1–29. 10.1111/j.1464-0597.2007.00325.x11845560

[11] Duan Y Li X Guo L Liang W Shang B Lippke S. Wechat mini program-based intervention for physical activity, fruit and vegetable consumption among chinese cardiovascular patients in home-based rehabilitation: a study protocol. Front Public Health. (2022) 10:739100. 10.3389/fpubh.2022.739100 35392478 PMC8980353

[12] Sun Y Fung SSW Man PKW Wan ANT Stewart S Lam TH. Promoting fruit and vegetable intake in parents: a cluster randomised controlled trial. Int J Environ Res Public Health. (2021) 18:5206. 10.3390/ijerph18105206 34068437 PMC8153553

[13] Dillon K Rollo S Prapavessis H. A combined health action process approach and mhealth intervention to reduce sedentary behaviour in university students - a randomized controlled trial. Psychol Health. (2022) 37:692–711. 10.1080/08870446.2021.1900574 33780297

[14] Asgari S Abbasi M Hamilton K Chen YP Griffiths MD Lin CY. A theory-based intervention to promote medication adherence in patients with rheumatoid arthritis: a randomized controlled trial. Clin Rheumatol. (2021) 40:101–11. 10.1007/s10067-020-05224-y 32588274 PMC7782392

[15] Schwarzer R Lippke S Luszczynska A. Mechanisms of health behavior change in persons with chronic illness or disability: the health action process approach (Hapa). Rehabil Psychol. (2011) 56:161–70. 10.1037/a0024509 21767036

[16] Wiener ES Mullins CD Pincus KJ. A framework for pharmacist-assisted medication adherence in hard-to-reach patients. Res Social Adm Pharm. (2015) 11:595–601. 10.1016/j.sapharm.2014.11.010 25638746

[17] Palinkas LA Horwitz SM Green CA Wisdom JP Duan N Hoagwood K. Purposeful sampling for qualitative data collection and analysis in mixed method implementation research. Adm Policy Ment Health. (2015) 42:533–44. 10.1007/s10488-013-0528-y 24193818 PMC4012002

[18] Creswel JW. Research Design: Qualitative, Quantitative, and Mixed Methods Approaches. 4th ed. City of Thousand Oaks, CA: SAGE Publications. (2014).

[19] Braun V Clarke V. Reflecting on reflexive thematic analysis. Qual Res Sport Exerc Health. (2019) 11:589–97. 10.1080/2159676X.2019.1628806

[20] Hallsworth K Dombrowski SU McPherson S Anstee QM Avery L. Using the theoretical domains framework to identify barriers and enabling factors to implementation of guidance for the diagnosis and management of nonalcoholic fatty liver disease: a qualitative study. Transl Behav Med. (2020) 10:1016–30. 10.1093/tbm/ibz080 31120519 PMC7543077

[21] Gu Y Zhou R Kong T Zhang W Chen Y Wang C. Barriers and enabling factors in weight management of patients with nonalcoholic fatty liver disease: a qualitative study using the COM-B model of behaviour. Health Expect. (2022) 26:355–65. 10.1111/hex.13665 36385729 PMC9854286

[22] Zhang XP Pan JH Wan LH Liu ZY Mo MM Wang MY. Factors influencing health behaviour, blood pressure control, and disability in hypertensive ischaemic stroke patients after a comprehensive reminder intervention. J Adv Nurs. (2020) 76:1384–93. 10.1111/jan.14340 32128865

[23] Chor D Pinho Ribeiro AL Sa Carvalho M Duncan BB Andrade Lotufo P Araujo Nobre A. Prevalence, awareness, treatment and influence of socioeconomic variables on control of high blood pressure: results of the Elsa-Brasil study. PLoS ONE. (2015) 10:e0127382. 10.1371/journal.pone.0127382 26102079 PMC4478044

[24] Newton JL. Systemic symptoms in nonalcoholic fatty liver disease. Dig Dis. (2010) 28:214–9. 10.1159/000282089 20460914

[25] Arora C Malhotra A Ranjan P Vikram NK Dwivedi SN Singh N. Perceived barriers and facilitators for adherence to lifestyle prescription: perspective of obese patients with non alcoholic fatty liver disease from North India. Diabetes Metab Syndr. (2021) 15:102138. 10.1016/j.dsx.2021.05.011 34186359

[26] Wang L Zhang J Liu Y Zhou H Yan W Ren H. The relationship between health-related fitness and quality of life in nonalcoholic fatty liver disease. Int J Environ Res Public Health. (2022) 19:14215 10.3390/ijerph192114215 36361098 PMC9654289

[27] Weinstein AA Escheik C Oe B Price JK Gerber LH Younossi ZM. Perception of effort during activity in patients with chronic hepatitis C and nonalcoholic fatty liver disease. PM R. (2016) 8:28–34. 10.1016/j.pmrj.2015.06.001 26071652

[28] Casey D De Civita M Dasgupta K. Understanding physical activity facilitators and barriers during and following a supervised exercise programme in type 2 diabetes: a qualitative study. Diabet Med. (2010) 27:79–84. 10.1111/j.1464-5491.2009.02873.x 20121893

[29] Francque SM Marchesini G Kautz A Walmsley M Dorner R Lazarus JV. Non-alcoholic fatty liver disease: a patient guideline. JHEP Rep. (2021) 3:100322. 10.1016/j.jhepr.2021.100322 34693236 PMC8514420

[30] Medicine ACoS. Acsm's Exercise Testing and Prescription. Philadelphia, PA: Lippincott Williams & Wilkins (2017).

[31] Avery L Exley C McPherson S Trenell MI Anstee QM Hallsworth K. Lifestyle behavior change in patients with nonalcoholic fatty liver disease: a qualitative study of clinical practice. Clin Gastroenterol Hepatol. (2017) 15:1968–71. 10.1016/j.cgh.2017.06.011 28624648

[32] Centis E Marzocchi R Di Domizio S Ciaravella MF Marchesini G. The effect of lifestyle changes in nonalcoholic fatty liver disease. Dig Dis. (2010) 28:267–73. 10.1159/000282101 20460922

[33] Tincopa MA Wong J Fetters M Lok AS. Patient disease knowledge, attitudes and behaviours related to nonalcoholic fatty liver disease: a qualitative study. BMJ Open Gastroenterol. (2021) 8:e000634. 10.1136/bmjgast-2021-000634 34193468 PMC8246278

