# Supplementary material for: Factors influencing adherence to lifestyle prescriptions among patients with nonalcoholic fatty liver disease: A qualitative study using the health action process approach framework
Source: Front Public Health. 2023 Mar 17;11:1131827. doi: 10.3389/fpubh.2023.1131827 (PMC10065407; doi:10.3389/fpubh.2023.1131827)
Supplement: Supplementary file 1 [file Table_1.DOCX]

Supplementary Material

**Table A: Consolidated criteria for reporting qualitative studies (COREQ) checklist.**

| **Domain 1 : Research team and reflexivity** | |
| --- | --- |
| **Personal characteristics** |  |
| 1. Interviewer | **Lina Wang; Xin Wang** |
| 2. Credentials | PhD, RN, MD |
| 3. Occupation | 1 Professor, 1 Nursing researcher, 2 Doctor |
| 4. Gender | 6 female, 1 male |
| 5. Experience & training | Lifestyle intervention for patients with fatty liver disease |
| **Relationship with participants** | |
| 6. Relationship established prior to  study commencement | No |
| 7. Participant knowledge of the  interviewer | Yes, reasons for doing research |
| 8. Interviewer characteristics | Reported in method part of the main document |
| **Domain 2: Study design** | |
| **Theoretical framework** |  |
| 9. Methodological orientation & theory | Reflexive thematic analysis and framework analysis |
| **Participant selection** |  |
| 10. Sampling | Purposive sampling |
| 11. Method of approach | Face to face interview |
| 12. Sample size | 30 in total |
| 13. Non-participation | None of the patients declined. |
| 14. Setting of data collection | In a separate room after patient’s outpatient visit |
| 15. Presence of non-participants | No |
| 16. Description of sample | Outlined in the main document (methods part) |
| **Data collection** |  |
| 17. Interview guide | Questions draft, piloted and revised |
| 18. Repeat interviews | No repeat interviews |
| 19. Audio/visual recording | Interviews were audio-recorded |
| 20. Field notes | Recorded after interviews |
| 21. Duration | Reported, range 10 to 40 minutes |
| 22. Data saturation | Sampling continued until data saturation |
| 23. Transcripts returned | Transcripts were available to participants on request |
| **Domain 3: analysis and findings** | |
| **Data analysis** |  |
| 24. Number of data coders | Outlined in the text, nine in total |
| 25. Description of coding tree | A coding tree was not developed |
| 26. Derivation of themes | Themes were derived from the data by thematic analysis |
| 27. Software | NVivo11 (QSR International, Melbourne, Australia) qualitative analysis software. |
| 28. Participant checking | No, the patient came to the hospitals were changing daily, unable to meet the same patients during the research period. |
| **Reporting** |  |
| 29. Quotations presented | Supporting quotations presented |
| 30. Data and findings consistent | Yes |
| 31. Clarity of major themes | A clear presentation of major themes is outlined |
| 32. Clarity of minor themes | Variations in views and themes and minor themes are  presented |
