# Supplementary material for: Factors influencing adherence to lifestyle prescriptions among patients with nonalcoholic fatty liver disease: A qualitative study using the health action process approach framework
Source: Front Public Health. 2023 Mar 17;11:1131827. doi: 10.3389/fpubh.2023.1131827 (PMC10065407; doi:10.3389/fpubh.2023.1131827)
Supplement: Supplementary file 2 [file Table_2.DOCX]

**Interview Guide**

1. Do you know about nonalcoholic fatty liver disease? What effect will it have on the body？
2. Do you know the effect of lifestyle change on the treatment of NAFLD? How did you learn about it?
3. Can you tell me what you know about how patients with fatty liver should change their lifestyle?
4. What impact do you think the healthy lifestyle of regular exercise and proper diet may have on you? Have you ever felt the impact?
5. Do you think that unhealthy lifestyle may affect you? Have you ever felt the impact?
6. What exercises have you tried before? Have you made plans and goals? Have you interrupted or abandoned your exercise? What is the reason?
7. What kind of exercise plan would you like to get if a professional makes an exercise plan for you?
8. Are you confident to stick to the habit of regular exercise for a long time? What problems do you think you will encounter? Are you confident to overcome the above problems? How to do it?
9. What dietary changes have you tried before? Have you made plans and goals? Have you interrupted or abandoned healthy eating behaviors? What is the reason?
10. What kind of diet plan would you like to get if a professional makes a diet plan for you?
11. Are you confident to stick to the habit of healthy eating for a long time? What problems do you think you will encounter? Are you confident to overcome the above problems? How to do it?
12. Do you think the support of family, friends and doctors, etc. can help you stick to a healthy lifestyle? Can you tell me in detail what kind of support you want?
13. Do you have anything to add? If I have any questions, can I contact you again?
